# The effect of the CGRP receptor antagonist (MK-8825) on the response to trigeminal nociception in rodents

**DOI:** 10.1186/1129-2377-14-S1-P93

**Published:** 2013-02-21

**Authors:** M Romero-Reyes, S Akerman

**Affiliations:** 1 NYU College of Dentistry, USA; 2 University of California, San Francsico, USA

## Introduction

Several highly prevalent and debilitating conditions involve pain in the trigeminal distribution. Although effective treatments exist, for many patients these treatments are far from optimal. Therefore understanding the pathophysiology of pain in the craniofacial region is critical. CGRP plays an essential role in the process of peripheral and central sensitization in the trigeminovascular system. Release of CGRP contributes to the process of neurogenic inflammation generating allodynia and hyperalgesia, clinical characteristics in neuropathic, inflammatory and migraine pain.

## Objectives

To study the effect of the CGRP receptor antagonist, MK-8825, on responses to trigeminal nociception and trigeminal nucleus caudalis (TNC) activation, using a mouse model of trigeminal nociception.

## Methods

C57BL/6 mice received either a subcutaneous injection of the MK-8825 (70mg/kg) or vehicle alone, followed by an injection of 0.9% saline solution or complete Freund’s adjuvant (CFA) in their right masseter muscle, shown previously to produce nociceptive-specific specific grooming behaviors. Animals were video recorded for assessment of nociceptive behaviors for one hour. Mice were overdosed with isoflurane and perfused transcardially at 2 or 24 hours after the MK-8825 injection. The brainstem was removed the area of the TNC was prepared and reacted for Fos immunoreactivity to verify neuronal activation.

## Results

Mice that received MK-8825, with CFA injection in the masseter, spent significantly less time exhibiting nociceptive behaviors compared to those that received vehicle (P < 0.05). At 2 and 24hrs after CFA injection mice that received MK-8825 had a significantly lower level of Fos immunoreactivity in the ipsilateral TNC compared to those that received CFA and vehicle.

## Conclusion

MK-8825 decreases pain behaviors and neuronal activation in the TNC of mice with inflammatory pain in the trigeminal mandibular distribution. CGRP likely contributes to the pathophysiological mechanism of this pain response.

## Conflicts

Grant funding from MERCK Sharp & Dohme Corp,

This work has been supported in part by a research grant from the Investigator Initiated Studies Program of Merck Sharp & Dohme Corp and 2009 AHS/MRF Thomas E. Heftler Migraine Research Award. We want to thank Dr. Andrew Charles for helpful discussions.

## Conflicts

Dr. Marcela Romero has received grant funding from Merck Sharp & Dohme Corp to conduct this study. Dr. Simon Akerman has consulted for Merck Sharp & Dohme Corp and MAP Pharmaceuticals

